# A Mapped Locus on LG A6 of *Brassica juncea* Line Tumida Conferring Resistance to White Rust Contains a CNL Type R Gene

**DOI:** 10.3389/fpls.2019.01690

**Published:** 2020-01-08

**Authors:** Latika Bhayana, Kumar Paritosh, Heena Arora, Satish Kumar Yadava, Priyansha Singh, Divakar Nandan, Arundhati Mukhopadhyay, Vibha Gupta, Akshay Kumar Pradhan, Deepak Pental

**Affiliations:** ^1^ Department of Genetics, University of Delhi South Campus, New Delhi, India; ^2^ Centre for Genetic Manipulation of Crop Plants, University of Delhi South Campus, New Delhi, India

**Keywords:** *Brassica* species, molecular mapping, mustard, white rust disease, CNL type R genes

## Abstract

White rust, causal agent oomycete *Albugo candida*, is a significant disease of the cultivated *Brassica* species. The Indian gene pool lines of oilseed mustard, *Brassica juncea*, are highly susceptible to the pathogen. Resistance to *A. candida* has been reported in the east European gene pool lines of mustard and mapped to LG A4 in line Heera and LG A5 in line Donskaja-IV. A new resistance-conferring locus to *A. candida* isolate AcB1 has been mapped to LG A6 of *B. juncea* line Tumida—a Chinese vegetable type mustard using an F_1_DH mapping population that has been developed from a Tumida × Varuna (susceptible Indian gene pool line) cross. A molecular map containing 8,303 genic and GBS markers was used to map the resistance trait to an interval of 63.0 cM—70.8 cM on LG A6. Genome assemblies of Tumida and Varuna were used to find the genes present within the flanking markers discerned by genetic mapping. The most likely candidate gene in the mapped interval is *BjuA046215*, a CC-NBS-LRR (CNL) type R gene that encodes a protein with all the specific subdomains of the proteins encoded by such genes. Alleles of *BjuA046215* in Varuna and other lines of the Indian and the east European gene pools encode proteins that have truncated LRR domains. Analysis of the syntenic regions in some of the Brassicaceae genomes and phylogenetic analysis of CNL type R genes showed *BjuA046215* to be closely related to a recently described white rust resistance-conferring R gene *BjuWRR1* in *B. juncea* Donskaja-IV, both belonging to the CNL-D group of R genes. Related R genes in *Arabidopsis thaliana* confer resistance to another oomycete, *Peronospora parasitica*.

## Introduction

White rust caused by the biotrophic oomycete *Albugo candida* is a major disease of oilseed and vegetable crops belonging to the genus *Brassica* (Saharan and Verma, 1992; Kamoun et al., 2015). The disease appears as white pustules containing zoospores on the abaxial leaf surface of susceptible plants; these pustules become more prominent as the disease progresses (Meena et al., 2014; Saharan et al., 2014). The infection can spread systemically to the reproductive parts of the infected plant resulting in abnormal inflorescences, called ‘stagheads’ that contain oospores (Saharan et al., 2014). Stagheads usually are co-infected with downy mildew caused by oomycete *Peronospora* species (Bains and Jhooty, 1985; Cooper et al., 2002). The Indian gene pool lines of *Brassica juncea* (oilseed mustard) are highly susceptible to *A. candida* (Li et al., 2008; Panjabi-Massand et al., 2010; Awasthi et al., 2012) leading to significant yield losses (Lakra and Saharan, 1989; Saharan and Verma, 1992). However, resistance to white rust has been reported in the east European gene pool lines of oleiferous mustard (Panjabi-Massand et al., 2010; Awasthi et al., 2012; Arora et al., 2019). Breeding for resistance to white rust in mustard is of critical importance as in India alone the crop is grown in ~6 million hectares of land.

Loci conferring resistance to *A. candida* have been mapped in *Arabidopsis thaliana* (Borhan et al., 2001; Cevik et al., 2019) and in the *Brassica* species—*B. rapa* (AA) (Kole et al., 1996; Kole et al., 2002), *B. juncea* (AABB) (Prabhu et al., 1998; Panjabi-Massand et al., 2010) and *Brassica napus* (AACC) (Ferreira et al., 1995). We have earlier reported mapping of resistance-conferring loci in two east European gene pool lines of mustard—Donskaja-IV and Heera. Two different F_1_DH populations derived from the crosses—Varuna (susceptible) × Heera (resistant), and TM-4 (susceptible) × Donskaja-IV (resistant) were used for mapping the resistance-conferring loci. Both Varuna and TM4 belong to the Indian gene pool of mustard (Srivastava et al., 2001). Disease assays were carried out using a highly infectious *A. candida* isolate AcB1. In Heera, the resistance-conferring locus AcB1-A4.1 was mapped on LG A4, and in Donskaja-IV locus AcB1-A5.1 was mapped on LG A5 (Panjabi-Massand et al., 2010). The two loci have been introgressed into four major varieties grown extensively in India. The gene conferring resistance to *A. candida* in the locus AcB1-A5.1 was identified to be a CC-NBS-LRR (CNL) type R gene named as *BjuA5.WRR.a1* (in short—*BjuWRR1*) that conferred resistance to several isolates of *A. candida* collected from different locations in the mustard growing regions of India (Arora et al., 2019).

The vegetable types of mustard, mostly grown in China, constitute the third gene pool of *B. juncea* (Yang et al., 2016). We found Tumida, a Chinese vegetable type mustard, to be resistant to *A. candida* isolate AcB1. By using an F_1_DH population derived from Tumida (resistant) × Varuna (susceptible) cross, we have mapped another major locus on LG BjuA6 of *B. juncea* that is involved with resistance to *A. candida*. Genome assemblies of Tumida (Yang et al., 2016) and Varuna (Paritosh et al., 2019) were used to analyze the genes present in the mapped region. The region was found to contain a CC-NBS-LRR (CNL) type R gene. We report here the structure and the evolution of the resistance-conferring locus and the candidate R gene.

## Materials and Methods

### Mapping Population and Disease Phenotyping

**Figure 1:**
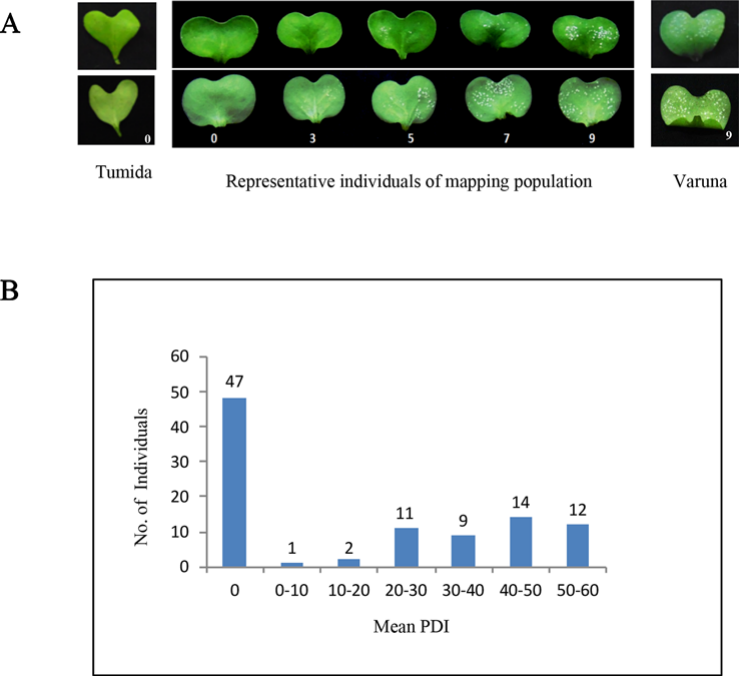
Disease reaction and mean percent disease index (PDI) of the parental lines and the TuV mapping population. **(A)** White rust disease reaction on the seedling cotyledons of the two parents and representative mapping lines illustrating the 0–9 scale used for scoring the infection. Tumida and mapping population lines showing no disease symptoms were given a score of 0, Varuna and lines having very high density of pustules on both the abaxial and adaxial surface were scored as 9, score 3 was given if there were one/few pustules on the abaxial/adaxial surface, score 5 was used if the pustule density was moderate and score 7 was used for high pustule density on the abaxial and a few pustules on the adaxial surface; **(B)** Frequency distribution of the TuV population lines based on the PDI score; 47 individuals were resistant having mean PDI = 0 whereas the remaining 49 lines were susceptible having mean PDI ≥ 1.

*B. juncea* lines—Tumida and Varuna were crossed to develop an F_1_DH (doubled haploid) mapping population (named TuV) by microspore culture following a protocol described earlier (Mukhopadhyay et al., 2007). The parental and the TuV DH population lines were maintained by strict self-pollination. A subset of 96 F_1_DH lines, randomly selected from a total of 750 DH lines, constituted the mapping population for genetic analyses and mapping of the disease resistance phenotype. *A. candida* isolate AcB1 (Panjabi-Massand et al., 2010) was used to screen for disease sensitivity/resistance. For infection assays, seeds were germinated in growth chambers set at a temperature of 22° ± 2°C, 10 h light/14 h dark cycle and 70% relative humidity (RH). Infection assays were carried out on 7-day old seedlings. Isolate AcB1 was maintained by repeated inoculations on a highly susceptible *B. juncea* line Varuna. Inocula for infections were prepared by scraping sporangia from infected cotyledons and suspending these in sterile double distilled water at a concentration of 5–10 × 10^4^ sporangia/ml of water. The suspension was kept on ice at 4°C for 3 h. Each cotyledon was drop inoculated with 20 µl of the suspension; infected seedlings were first kept in an infection chamber maintained at 18° ± 2°C and 90% RH for 24 h in the dark, and subsequently grown under 16 h light/8 h dark cycle for 10 days to score the extent of disease reaction. Infections were scored on a scale of 0–9, as illustrated in **Figure 1A**. In each experiment five seedlings of each of the two parents and F_1_DH lines were drop inoculated with isolate AcB1 and the disease phenotype was scored both as a qualitative (categorized as susceptible/resistant) and as a quantitative trait—measured as percent disease index (PDI) that was calculated following McKinney (1923) and Panjabi-Massand et al. (2010) using the formula: PDI = [Sum of numerical ratings/(number of cotyledons scored × maximum score) × 100]. Each experiment was repeated three times; mean PDI [mean percent disease index = (PDI1 + PDI2 + PDI3)/3)] was calculated for the parents, and each of the 96 lines.

### Construction of a Linkage Map and Mapping of the Resistance Trait

Genetic mapping was carried out using IP (Intron Polymorphism), genic SSR (simple sequence repeats) and genic SNP (assayed by KASP technology, USA) markers developed earlier for different *B. juncea* mapping populations in the laboratory (Panjabi et al., 2008; Paritosh et al., 2014; Dhaka et al., 2017). Further, GBS based markers described recently (Paritosh et al., 2019) were added to the map. In the first instance, an anchor map was developed with the IP, SSR and genic SNP markers using the mapping software JoinMap 4.1 (Van Ooijen, 2006) at a LOD threshold value greater than 5.0 using regression mapping. The Kosambi mapping function (Kosambi, 1944) was used to convert recombination frequencies into the genetic distance. In the second step, a high-density linkage map was developed by combining the anchor markers with GBS-based SNPs using the ASMap package (Taylor and Butler, 2017) which uses MSTmap algorithm (Wu et al., 2008) for linkage group construction. The package was used with the default parameters except for a few changes—the probability value (p.value) was set to 1e−15, and the missing threshold (miss.thresh) value to 0.3. A total of 18 linkage groups (LGs) referred to as BjuA1–BjuA10 and BjuB1–BjuB8, as described previously (Paritosh et al., 2019) were identified. Mapchart 2.2 (Voorrips, 2002) was used for a graphical representation of LG BjuA6.

QTL mapping was performed using the software package MapQTL6 (Van Ooijen and Kyazma 2009) using Kruskal–Wallis, interval mapping, and Multiple QTL Mapping (MQM) algorithms. LOD thresholds for QTL significance were determined for each LG by taking the number of permutations to be 1,000 and a significance level of 0.05. The nonparametric mapping using Kruskal–Wallis and interval mapping was performed prior to MQM mapping to identify closely linked markers. The markers which were close to the QTL and were identified both in the Kruskal–Wallis test and interval mapping were used as cofactors in MQM mapping. The marker closest to the peak LOD was identified, and the corresponding flanks were determined subsequently.

### Physical Mapping, Gene Characterization, and Synteny Analysis

Genomic sequences between the flanking markers in the mapped interval were downloaded from the BRAD database (Cheng et al., 2011). Genes annotated in *B. juncea* Tumida were taken from the genome assembly of the line (Yang et al., 2016). BRAD database was used for obtaining the sequence information of the syntenic regions in *A. thaliana*, *B. rapa*, and other sequenced *Brassica* species. The sequences of the genes present in Varuna were obtained from an SMRT based gene assembly of *B. juncea* Varuna (Paritosh et al., 2019). The nucleotide sequences of the resistant/susceptible alleles were compared using the CLUSTALW algorithm of MegAlign (DNASTAR, USA). The gene structure and the encoded protein sequences were represented by Snapgene viewer (GSL Biotech, USA). The structure of allelic variants in different lines of *B. juncea* and the synteny analysis was plotted using R programming.

For expression analysis, RNA was extracted from the plant tissues with ‘Spectrum Plant Total RNA Kit’ (Sigma-Aldrich, USA) and treated with DNaseI (Qiagen, Netherlands) to remove any genomic DNA contamination. 2 µg of total RNA was used to synthesize the cDNA with ‘High-Capacity cDNA Reverse Transcription Kit’ (Applied Biosystems, USA). The ubiquitin gene, UBQ9 (Chandna et al., 2012) was used as an internal control. cDNA was amplified with ‘Phusion High-Fidelity DNA Polymerase’ (Thermo Fisher Scientific, USA). Genomic DNA and cDNA were sequenced on an ABI PRISM sequencer (Applied Biosystems, USA).

### Phylogenetic Analysis

Protein sequences of NBS type genes present in *A. thaliana* and *B. rapa* that belonged to the CNL clade (Yu et al., 2014) were downloaded from TAIR database (Rhee et al., 2003) and BRAD database. For the alignment of the NBS domain, complete predicted protein sequences were trimmed at ten amino acids N terminal to the first Gly in the P-loop motif and 30 amino acids beyond the MHDV motif. Sequences were then aligned with the MUSCLE program, available in MEGA7 (Kumar et al., 2016) using default options. A phylogenetic tree was constructed using the Maximum Likelihood method based on the JTT matrix-based model (Jones et al., 1992). All positions with less than 70% site coverage were eliminated, and the reliability of the interior nodes was assessed using 1,000 bootstrap replicates.

## Results

### Disease Reaction of the TuV Population and Genetic Mapping of the Resistance Trait

Tumida was assigned a score of 0 as it was completely resistant to *A. candida* isolate AcB1 and showed no pustule formation in repeated experiments. In comparison, Varuna was allocated a score of 9 (maximum score), as it showed very high pustule density both on the abaxial and adaxial surfaces (**Figure 1A**). Out of the 96 lines of the TuV population, 47 were observed to be completely resistant, and 49 were observed to be susceptible to varying degrees (**Figure 1B**, **Supplementary Table S1**). The resistant/susceptible lines in the mapping population approached a 1:1 ratio (χ^2^ = 0.04, d.f = 1, p <0.05), characteristic of monogenic inheritance of a trait. The segregation pattern strongly suggested the presence of a single major locus in Tumida conferring resistance to the white rust disease.

A genetic linkage map was developed using a set of 96 DH lines of the TuV population. To develop the molecular map—1,980 IP, 2,200 genic SSR, 1,175 genic SNP markers earlier used for mapping in different *B. juncea* bi-parental populations were screened on Tumida and Varuna for identifying markers that were polymorphic between the two parents. The screening identified 535 polymorphic markers (**Supplementary Table S2**), which were used to develop a framework map. The map had a total length of 1,410.5 cM with an average marker spacing of 2.5 cM. However, the linkage map contained significant gaps in almost all the LGs. GBS based SNP markers (Paritosh et al., 2019) were used for further saturation of the TuV map.

A total of 7,786 unambiguous GBS-based SNPs, with missing data less than 30%, were added to the anchor markers for developing a high-density linkage map (**Supplementary Table S2**). The final TuV genetic map had a total length of 2,660.6 cM with an average marker spacing of 0.4 cM. The number of markers in the eighteen linkage groups varied from 106 (BjuA4) to 910 (BjuB3); the genetic lengths spanned in each of the 18 linkage groups ranged from 74.1 (BjuA4) to 236.0 cM (BjuB8). The marker density (number of markers/cM) that was 0.4 in the anchor map was improved to 3.0 in the final map (**Supplementary Table S3**).

The resistance trait was first mapped qualitatively. Individual lines of the mapping population were scored as either resistant or susceptible (“a” or “b”). The lines that were consistently free of pustules were marked as resistant whereas those that had any pustule were marked as susceptible. The resistant trait mapped on LG BjuA6 and co-segregated with the GBS based marker rs377790.

**Figure 2:**
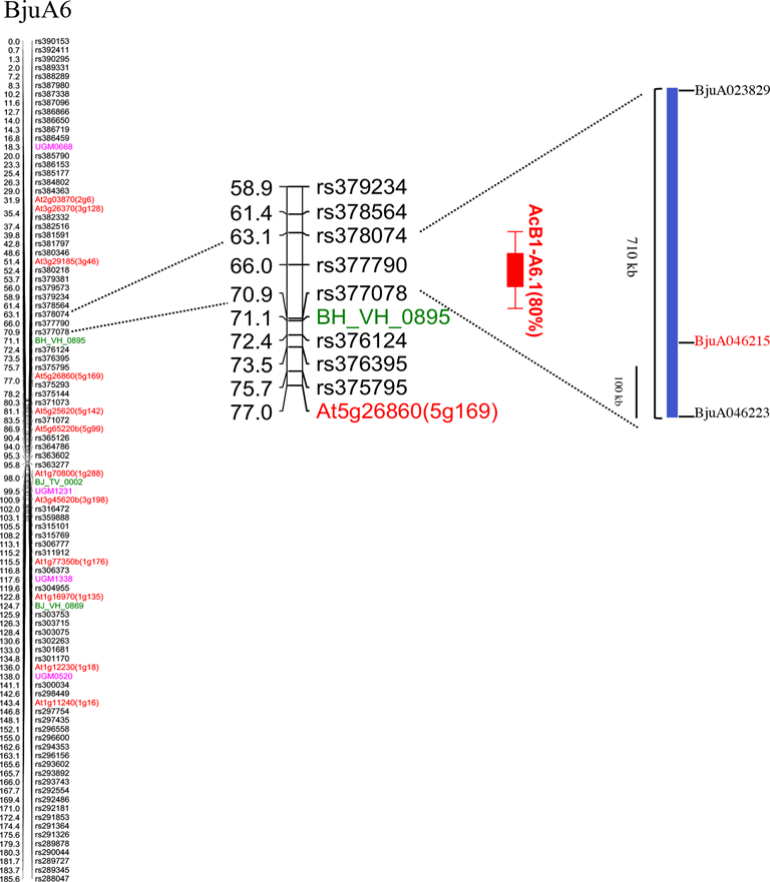
Mapping of white rust resistance in the LG BjuA6. The LG contained 629 polymorphic markers (markers mapping at the same position have been removed). The genic SSR, IP, genic SNPs, and GBS based SNPs are represented with pink, red, green and black color, respectively. A single major QTL (AcB1-A6.1; red bar) conferring resistance to white rust isolate AcB1 mapped to an interval of 63.0–70.8 cM. A CNL type R gene, *Bju046215*, was identified in the Tumida genomic sequence spanning the QTL region.

We also carried out QTL mapping of the resistance trait using the PDI scores (**Figure 1B**) to discern the contribution of the locus mapped with qualitative R/S scoring and to map any other loci contributing to resistance. The resistance phenotype mapped as a single major QTL on LG BjuA6 and was named AcB1-A6.1 following an earlier convention (Panjabi-Massand et al., 2010). This major QTL mapped in the genetic interval of 63.0–70.8 cM and was flanked by markers—rs377078 and rs378074 with a peak LOD value of 33.5. The peak LOD value was substantially higher than the genome-wide LOD significance threshold value of 3.5 determined by permutation testing with 1,000 replicates. The AcB1-A6.1 QTL explained 80% of the total phenotypic variance with an additive effect of −19.6%. The negative effect implied that the Varuna allele controlled the disease severity. The closest linked marker with the disease trait showing the highest LOD value was rs377790 which had earlier shown co-segregation with resistance in the qualitative mapping (**Figure 2**).

### Gene Content Analysis in the AcB1-A6 Locus in Tumida and Varuna and Syntenic Regions in *B. rapa and A. thaliana*

Gene sequences between the flanking markers rs377078 and rs378074, covering a genetic interval of 7.8 cM, were identified in the genome assemblies of *B. juncea*—Tumida and Varuna, *B. rapa* and *A. thaliana* based on gene collinearity. The flanking markers encompassed a 710 kb region in the Tumida genome assembly that contained 50 genes. In Varuna—a 344 kb region with 63 genes was identified. Forty-one of the 50 genes, identified in Tumida genome assembly, had orthologs in *A. thaliana*. All the predicted genes in the mapped interval in *A. thaliana*, *B. rapa*, and *B. juncea—*Tumida and Varuna were listed and compared (**Supplementary Table S4**). Information on the predicted molecular/biological function of the genes in *A. thaliana* was taken from the TAIR database. It was found that a few of the listed genes had been predicted to play a secondary role in defense; none of these had been functionally validated in *A. thaliana.* However, we could identify an R gene (*BjuA046215*) belonging to the CC-NBS-LRR (CNL) class in the mapped interval of *B. juncea* Tumida. The marker rs377790, which was co-segregating with the phenotypic marker in genetic mapping, was found to be in the gene itself. Orthologs of the R gene were found to be present in the syntenic genomic region of *B. rapa* Chiifu and *B. juncea* Varuna but absent in *A. thaliana* (**Supplementary Table S4**). *BjuA046215* was considered as the most likely candidate gene involved with conferring resistance to white rust disease and was analyzed further.

### Gene Structure of *BjuA046215* in Tumida and Other *B. juncea* Lines

**Figure 3:**
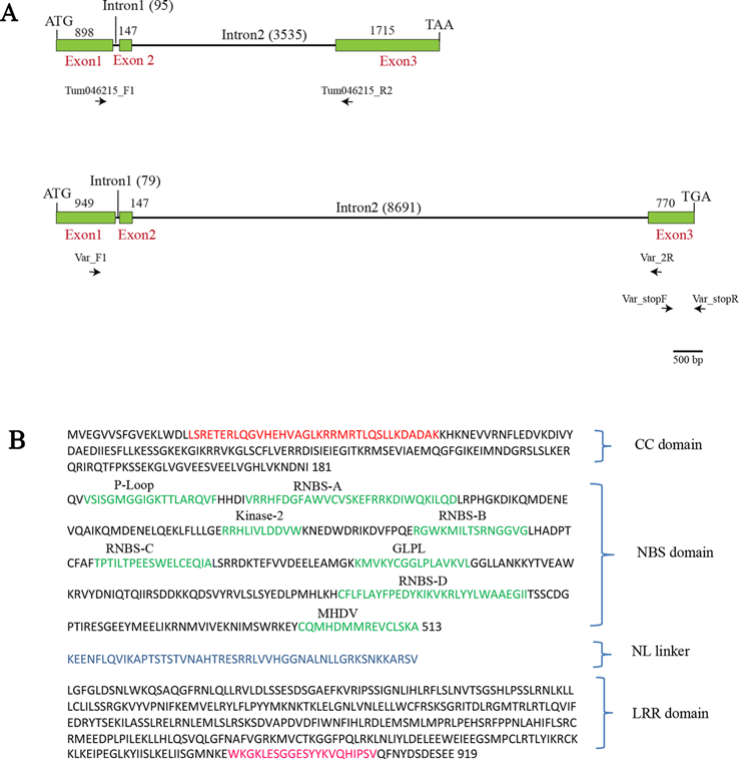
The overall structure of *BjuA046215* gene in the parental lines—Tumida (resistant) and Varuna (susceptible) and the encoded protein sequence in Tumida. **(A)** Structure of the resistant and susceptible alleles; ATG and TAA are the start and stop codons in the resistant parent Tumida, whereas ATG and TGA are the start and the stop codons in the susceptible parent Varuna. The gene-specific forward and reverse primers—Var_F1, Var_2R, Tum046215_F1, Tum046215_R2 were used for expression analysis of Tumida and Varuna allele and the primer pair Var_stopF and Var_stopR were used for validating the nucleotide sequence duplication in the Exon 3 of Varuna leading to a premature stop codon. **(B)** The encoded polypeptide of Tumida allele had all the conserved motifs of the CC-NBS-LRRs class of R genes. The conserved motifs in the coiled-coil (CC) domain, NBS domain, NL linker, and LRR domain are shown in red, green, blue, and pink colors, respectively.

*BjuA046215* gene was 6.39 kb long having three exons and two introns. Exon 1 (E1) was 898 bp, Exon 2 (E2) was 147 bp, and Exon 3 (E3) was 1715 bp in length. E1 and E2 were interrupted with 95 bp long intron 1 whereas E2 and E3 were interrupted with a 3,535 bp long intron 2. We found the presence of a non-canonical GC splice site at the 5’ end of Intron 1 instead of the commonly encountered canonical dinucleotide GT. However, the non-canonical splice site was confirmed to be functional by sequencing of the genomic DNA and the cDNA (**Supplementary Figure S1**). Such non-canonical sites have been reported to be functional in some other genes also (Pucker and Brockington, 2018). *BjuA046215* had a coding region of 2,760 bp and encoded a protein of 919 amino acid residues with an estimated molecular weight of 106.5 kDa (**Figure 3A**). We analyzed the BjuA046215 protein with the domain prediction software InterProscan (Finn et al., 2017); the encoded protein had all the conserved motifs like CC motif, P-loop, RNBS-A, B, C, D, MHDV, GLPL and Kinase 2, NL linker and LRR motifs which define the CC, NB and LRR domains of the CNL type of NBS-LRRs (Meyers et al., 2003) (**Figure 3B**).

The allelic variant of *BjuA046215* in Varuna (reported as gene A06_g5020 by Paritosh et al., 2019) was 10.63 kb in size having an overall similarity in structure to the Tumida allele. E1 was 949 bp, E2 was 147 bp, and E3 was 770 bp long interrupted by two introns of size 79 bp and 8.69 kb, respectively (**Figure 3A**). The gene had a coding region of 1,866 bp. The CDS of Varuna gene had 94.45% sequence identity and 91.79% protein similarity (NCBI blastn and blastp) with the Tumida gene. A duplicated five bp sequence (TTTAA) was found to be present at the position 755–760 in Exon 3 of Varuna allele (**Supplementary Figure S2**) resulting in a premature stop codon in the ORF. The finding was validated with region-specific primers, spanning the site of the duplication (**Figure 3A**, **Supplementary Table S5**) by three independent PCR reactions (**Supplementary Figure S2**). The mutant allele in Varuna encoded a protein of 621 amino acid residues with truncation in the LRR region (**Supplementary Figure S3**).

**Figure 4:**
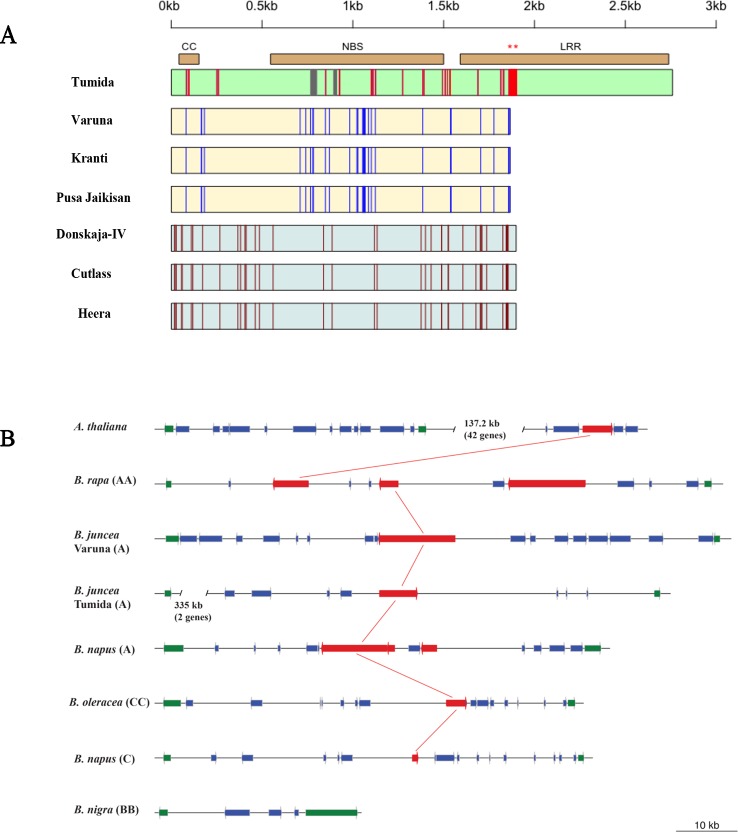
Allelic diversity in the R gene *BjuA046215* in the sequenced genome assemblies of some *B. juncea* germplasm and its orthologs in the syntenic regions across *Brassica* species and *A. thaliana*. **(A)** Allelic structure variants of *BjuA046215* gene—boxes represent the CDS region drawn from the start to stop codon. The Indian, east European and Chinese allelic variants have been represented by yellow, grey, and green color, respectively. Allele specific SNPs have been shown by red, blue and brown colored lines whereas grey colored vertical lines represent deletions. Two different mutations leading to premature stop codons were observed in the LRR regions of the Indian and east European gene pool lines—shown by asterisks on the top of the horizontal bar representing the LRR region. **(B)** The orthologs of *BjuA046215* gene in *A. thaliana* and sequenced members of the *Brassica* species—the R genes have been represented in red and the genes defining the syntenic boundary have been shown in green, the other genes have been shown in blue; the most related orthologs of the CNL type R gene in Tumida have been connected with red color lines.

We also looked at the nucleotide sequences of the alleles of *BjuA046215* in different lines of *B. juncea* belonging to the Indian and east European gene pools. These lines have been recently sequenced in the lab (unpublished results). Three different allelic variants—the Indian, east European, and Chinese could be observed (**Figure 4A**). The Indian gene pool lines (allelic variant 1)—Kranti and Pusa Jaikisan had premature truncation in the LRR region at the same position as in Varuna, leading to a loss of 298 amino acids. Donskaja-IV, Cutlass, and Heera also had a premature truncation in the LRR encoding region; however, the site of the truncation was different from the one observed in the Indian gene pool lines. This truncation caused a loss of 286 amino acids. The three east European gene pool lines constituted the second allelic variant. The third allele is of Tumida, the Chinese vegetable type gene pool line, which seemed to contain the complete LRR region and therefore, could be functional in conferring resistance to the white rust disease. Expression of the R gene in Tumida and Varuna was checked by synthesizing cDNA, which was used as a template for PCR amplification with gene-specific primers designed from the intron spanning exons (**Supplementary Table S5**). Both Tumida and Varuna genes expressed constitutively, in the absence of any infection.

### Evolutionary Relationship of *BjuA046215* With CNL Type Genes *in Brassica* Species and *A. thaliana*

The syntenic regions of some of the sequenced *Brassica* species and *A. thaliana* were studied to locate orthologs of the R gene, *BjuA046215*. We could find one CNL type R gene (*At5g48620*) in *A. thaliana* that was not present in the syntenic region but was located 137.2 kb away from the region (**Figure 4B**). The syntenic region in *B. rapa* (Chiifu) contained three paralogs—*Bra037453, Bra037451, and Bra037448.* Like in *B. rapa*, the *B. napus* (A genome) also contained three paralogs of the R gene. Only one CNL type R gene was identified in the A genome of *B. juncea* lines—Varuna and Tumida. *B. oleracea* and *B. napus* (C genome) also contained a single ortholog of *BjuA046215*. Syntenic region of *B. nigra* (BB) did not contain any CNL type R gene (**Figure 4B**).

**Figure 5:**
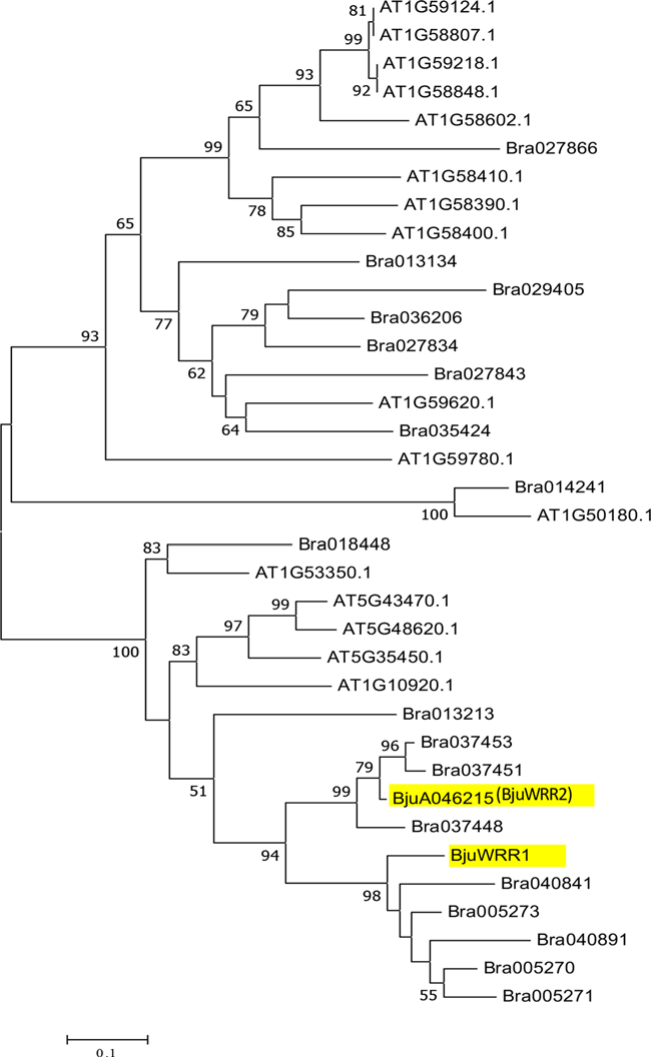
Phylogenetic relationship of NBS-encoding genes belonging to the CNL-D subgroup in *A. thaliana*, *B. rapa*, and the genes described in *B. juncea—*(*BjuWRR1)* and *BjuA046215* (*BjuWRR2*) that confer resistance to *Albugo candida*.

We assessed sequence similarity/diversity and relationship of the candidate gene *BjuA046215* with the well-characterized white rust resistance-conferring gene *BjuWRR1* (Arora et al., 2019) and the genes present in *A. thaliana* and *B. rapa* belonging to the CNL clade (Yu et al., 2014). Phylogenetic analysis carried out as described in the materials and methods section revealed the presence of four subgroups—CNL-A, CNL-B, CNL-C and CNL-D (**Supplementary Figure S4**) as reported earlier (Meyers et al., 2003). The candidate gene, *BjuA046215*, was found to be present in the CNL-D subgroup of the phylogenetic tree along with *BjuWRR1*, a CNL type R gene identified in *B. juncea* Donskaja-IV on LG A5 (Arora et al., 2019) (**Figure 5**).

## Discussion

We have mapped a novel locus in *B. juncea* Tumida that confers resistance to white rust disease caused by *A. candida*. The resistance-conferring locus AcB1-A6.1 mapped to LG BjuA6 and is additional to the two loci mapped earlier—AcB1-A4.1 in Heera and AcB1-A5.1 in Donskaja-IV, the two lines belonging to the east European gene pool of mustard (Panjabi-Massand et al., 2010). The locus AcB1-A6.1 contains a CC-NBS-LRR (CNL type R gene) described as *BjuA046215* in the *B. juncea* Tumida genome assembly (Yang et al., 2016). This R gene is the most likely candidate gene that could be involved with conferring resistance to the isolate AcB1. *BjuA046215* has a structure that is typical of CC-NBS-LRR class of R type genes and an inheritance pattern of a single dominant gene usually encountered in effector-triggered immunity (ETI) based on R-Avr interaction (Jones and Dangl, 2006; Cevik et al., 2019). *BjuA046215* is phylogenetically related to the CNL type R gene *BjuWRR1*, identified in the east European gene pool line Donskaja-IV that has been shown to confer resistance to a number of white rust isolates including AcB1 (Arora et al., 2019). *BjuA046215* can, therefore, be named as *BjuA6.WRR.b1* (in short *BjuWRR2*) as it is the second R gene found to confer resistance to *A. candida* in *B. juncea*.

The most extensive work on the R genes involved with resistance to *A. candida* has been carried out in *A. thaliana* (Borhan et al., 2004; Borhan et al., 2008; Cevik et al., 2019). *A. thaliana* is not a natural host of *A. candida—*therefore, *A. thaliana* mutant line Ws-2-*eds1* and two transgressive segregants identified for susceptibility from a MAGIC population, have been used for identifying resistance-conferring genes (Cevik et al., 2019) in this model plant species. All the identified genes—*RAC1*, *WRR4* (Borhan et al., 2004; Borhan et al., 2008), *WRR4B*, *WRR8*, *WRR9* and *WRR12* (Cevik et al., 2019) belong to the TNL class of NBS-LRR genes. Till now, only two CNL type R genes, *BjuWRR1* (Arora et al., 2019) and *BjuWRR2* (this study) conferring resistance to *A. candida* have been identified; both the genes belong to the CNL-D subgroup. Phylogenetically, the most related gene(s) to the two *B. juncea* genes in *A. thaliana* are *AT5G48620*, *AT5G43470*, *AT5G35450*, and *AT1G10920*. *AT5G43470* has been shown to have evolved from *AT5G48620* by duplication and transposition of a region containing the latter gene (Meyers et al., 2003). *AT5G43470* is a well-characterized gene that confers resistance to some viral pathogens and an oomycete—*Peronospora parasitica* (Cooley et al., 2000; Takahashi et al., 2002), which causes downy mildew in *A. thaliana* and other species of Brassicaceae; no function has been assigned so far to *AT5G48620*. The other R genes belonging to the CNL-D lineage in *A. thaliana* have also been shown to confer resistance to *P. parasitica* (Eulgem et al., 2007). It may be that the related CNL-D lineage R genes have been recruited in *A. thaliana* ecotypes for resistance to oomycete *P. parasitica* and in *Brassica* species for conferring resistance to the more predominant oomycete pathogen *A. candida*.

Analysis of allelic variants of *BjuA046215* in the Indian (Varuna, Pusa-Jaikisan and Kranti), east European (Donskaja- IV, Cutlass, and Heera) and Chinese (Tumida) gene pool lines suggested the presence of three types of alleles. A clear correlation between the gene pool and the alleles could be observed—Allele 1 present in the Chinese vegetable type Tumida encodes for a complete CNL protein with all the three domains—(CC, NB and LRR), whereas mutations at distinct positions leading to truncation of the LRR domain were observed in the Indian (Allele 2) and the east European gene pools (Allele 3). An analysis of the syntenic regions in the genome assemblies of the *Brassica* species belonging to U’s Triangle (Nagaharu, 1935), showed the presence of one or more orthologs—three paralogs in *B. rapa* (AA) and the A genome of *B. napus* and one ortholog in *B. oleracea* (CC) and *B. napus* C genome as well as in the *B. juncea* A genome. Orthologs identified in other *Brassica* species have not been tested for resistance to *A. candida*. Variability studies in *B. juncea* germplasm have found significantly higher morphological and genetic diversity amongst Chinese vegetable type mustards in comparison to the germplasm from the other regions (Wu et al., 2009; Chen et al., 2013; Yang et al., 2018). It will be useful to look at the allelic diversity for *BjuWRR2* in the diverse vegetable and their related oil-yielding types of mustard from the Chinese gene pool of *B. juncea* to identify more R genes that could be deployed in future to impart durable and broad-spectrum resistance to *A. candida*. Orthologs of *BjuWRR2* in the syntenous regions of the related *Brassica* species could also be tested for their potential to confer resistance to white rust in *B. juncea*.

## Data Availability Statement

The sequence reads have been deposited under NCBI bioproject PRJNA550308.

## Author Contributions

LB and DN did the disease assays. LB, SY, PS, and VG carried out the mapping work. AM developed the mapping population. KP carried out genome sequencing and analysis. HA did the phylogenetic analysis. LB, KP, and HA did the bioinformatics-based analysis. AP and DP conceived the study. LB and DP wrote the paper.

## Funding

The study was supported by the Department of Biotechnology (DBT), Government of India through the award of three projects—an India–UK project (BT/IN/Indo-UK/CGAT/12/DP/2014-15), Centre of Excellence on Genome Mapping and Molecular Breeding of Brassicas (BT/01/COE/08/06/-II) and DBT-UDSC Partnership Centre on Genetic Manipulation of Brassicas (BT/01/NDDB/UDSC/2016).

## Conflict of Interest

The authors declare that the research was conducted in the absence of any commercial or financial relationships that could be construed as a potential conflict of interest.
