# Supplementary material for: A Mapped Locus on LG A6 of Brassica juncea Line Tumida Conferring Resistance to White Rust Contains a CNL Type R Gene
Source: Front Plant Sci. 2020 Jan 8;10:1690. doi: 10.3389/fpls.2019.01690 (PMC6960627; doi:10.3389/fpls.2019.01690)
Supplement: Supplementary file 1 [file DataSheet_1.pdf]

Table S1. Disease phenotype and mean PDI score for the two parents and TuV F_1DH lines

S.no.	Line number	Phenotype	Mean PDI
Parent1	Tumida	Resistant	0.0
Parent2	Varuna	Susceptible	67.6
1.	TUV-10	Susceptible	50.0
2.	TUV-17	Resistant	0.0
3.	TUV-22	Susceptible	17.0
4.	TUV-40	Susceptible	35.3
5.	TUV-43	Resistant	0.0
6.	TUV-46	Resistant	0.0
7.	TUV-47	Susceptible	33.7
8.	TUV-50	Susceptible	29.4
9.	TUV-51	Resistant	0.0
10.	TUV-55	Susceptible	25.9
11.	TUV-61	Susceptible	2.2
12.	TUV-64	Susceptible	26.8
13.	TUV-68	Susceptible	55.0
14.	TUV-72	Resistant	0.0
15.	TUV-75	Susceptible	51.5
16.	TUV-76	Susceptible	24.9
17.	TUV-78	Resistant	0.0
18.	TUV-80	Resistant	0.0
19.	TUV-83	Susceptible	37.4
20.	TUV-88	Susceptible	34.1
21.	TUV-91	Susceptible	47.1
22.	TUV-93	Susceptible	23.8
23.	TUV-94	Resistant	0.0
24.	TUV-98	Susceptible	18.5
25.	TUV-102	Susceptible	38.8
26.	TUV-104	Resistant	0.0
27.	TUV-107	Susceptible	26.5
28.	TUV-109	Resistant	0.0
29.	TUV-110	Susceptible	59.2
30.	TUV-117	Resistant	0.0
31.	TUV-119	Resistant	0.0
32.	TUV-120	Susceptible	42.6
33.	TUV-123	Susceptible	54.2
34.	TUV-135	Resistant	0.0
35.	TUV-149	Susceptible	56.5
36.	TUV-152	Susceptible	44.5
37.	TUV-153	Resistant	0.0
38.	TUV-164	Resistant	0.0
39.	TUV-169	Susceptible	46.7
40.	TUV-180	Susceptible	28.9

41.	TUV-183	Resistant	0.0
42.	TUV-186	Resistant	0.0
43.	TUV-188	Resistant	0.0
44.	TUV-191	Resistant	0.0
45.	TUV-194	Resistant	0.0
46.	TUV-201	Susceptible	53.3
47.	TUV-203	Susceptible	41.8
48.	TUV-209	Resistant	0.0
49.	TUV-212	Resistant	0.0
50.	TUV-213	Susceptible	29.7
51.	TUV-214	Resistant	0.0
52.	TUV-215	Resistant	0.0
53.	TUV-216	Susceptible	47.8
54.	TUV-218	Susceptible	20.4
55.	TUV-220	Susceptible	25.3
56.	TUV-223	Resistant	0.0
57.	TUV-225	Susceptible	39.2
58.	TUV-226	Susceptible	34.1
59.	TUV-227	Susceptible	37.8
60.	TUV-229	Susceptible	25.8
61.	TUV-231	Resistant	0.0
62.	TUV-232	Resistant	0.0
63.	TUV-233	Resistant	0.0
64.	TUV-234	Resistant	0.0
65.	TUV-235	Resistant	0.0
66.	TUV-239	Susceptible	43.4
67.	TUV-240	Resistant	0.0
68.	TUV-241	Susceptible	48.7
69.	TUV-244	Resistant	0.0
70.	TUV-245	Resistant	0.0
71.	TUV-248	Susceptible	37.0
72.	TUV-249	Susceptible	56.2
73.	TUV-250	Resistant	0.0
74.	TUV-254	Resistant	0.0
75.	TUV-256	Resistant	0.0
76.	TUV-259	Resistant	0.0
77.	TUV-263	Susceptible	47.4
78.	TUV-265	Susceptible	50.1
79.	TUV-268	Resistant	0.0
80.	TUV-269	Resistant	0.0
81.	TUV-271	Resistant	0.0
82.	TUV-274	Resistant	0.0
83.	TUV-277	Resistant	0.0
84.	TUV-279	Resistant	0.0
85.	TUV-280	Resistant	0.0
86.	TUV-281	Resistant	0.0
87.	TUV-283	Resistant	0.0
88.	TUV-287	Susceptible	47.0

89.	TUV-288	Susceptible	44.3
90.	TUV-290	Resistant	0.0
91.	TUV-292	Susceptible	57.3
92.	TUV-294	Susceptible	52.9
93.	TUV-296	Susceptible	51.8
94.	TUV-297	Susceptible	44.1
95.	TUV-298	Susceptible	48.3
96.	TUV-299	Susceptible	56.7

Table S2. Type and number of different molecular markers used for the construction of a linkage map in *B. juncea* TuV population

S.No.	Type of markers	Total number of markers tested*	Number of polymorphic markers	Number of markers mapped in the TuV linkage map
1.	IP (Intron length polymorphism)	1,980	186	169
2.	SSR (genic)	2,200	179	161
3.	Genic SNP (Single nucleotide polymorphism)	1,175	205	187
4.	GBS (Genotype by sequencing) (Newly developed for TuV population)	8,390	8,311	7,786
5.	Total number	13,745	8,881	8,303

* Markers were taken from the following studies in the laboratory-

1. IP markers (Panjabi et.al. 2008)
2. Genic SSRs (Dhaka et al. 2017)
3. Genic SNPs (Paritosh et al. 2014)
4. GBS based SNPs (Paritosh et al. 2019)

Table S3. Features of the genetic map of TuV F_1DH – framework map and the final map containing both the anchor markers and the GBS markers

LG	Length (cM)		No. of Markers		No. of marker intervals		Average Interval size (cM)		Marker Density	
	TuV. anchor	TuV. final	TuV. anchor	TuV. final	TuV. anchor	TuV. final	TuV. anchor	TuV. final	TuV. anchor	TuV. final
BjuA1	84.9	111.0	43	375	42	68	2.0	0.3	0.5	3.4
BjuA2	35.1	85.4	25	399	25	47	1.4	0.2	0.7	4.7
BjuA3	86.7	126.3	38	504	37	70	2.3	0.3	0.4	4.0
BjuA4	58.8	74.1	19	106	19	39	3.1	0.7	0.3	1.4
BjuA5	65.3	165.6	26	634	26	91	2.5	0.3	0.4	3.8
BjuA6	61.3	185.6	32	629	32	95	1.9	0.3	0.5	3.4
BjuA7	88.0	111.4	25	359	25	49	3.5	0.3	0.3	3.2
BjuA8	81.6	124.9	28	501	28	71	2.9	0.2	0.3	4.0
BjuA9	65.6	203.5	31	687	31	104	2.1	0.3	0.5	3.4
BjuA10	73.5	113.6	26	314	26	53	2.8	0.4	0.4	2.8
**∑ (A genome)**	700.8	1301.4	293	4508	291	687	-	-	-	-
**Mean**	70.1	130.1	29.3	450.8	29.1	68.7	2.5	0.3	0.4	3.41
BjuB1	57.2	135.3	29	498	28	67	2.0	0.3	0.5	3.7
BjuB2	71.5	104.7	34	449	33	50	2.2	0.2	0.5	4.3
BjuB3	90.1	208.6	53	910	53	118	1.7	0.2	0.6	4.4
BjuB4	86.4	203.3	29	443	29	90	3.0	0.5	0.3	2.2
BjuB5	114.5	141.4	22	155	22	44	5.2	0.9	0.2	1.1
BjuB6	76.3	145.4	24	382	24	76	3.2	0.4	0.3	2.6
BjuB7	114.8	184.4	23	430	23	86	5.0	0.4	0.2	2.3
BjuB8	98.9	236.0	28	528	28	111	3.5	0.4	0.3	2.2
**∑ (B genome)**	709.8	1359.1	242	3795	240	642	-	-	-	-
**Mean**	88.7	169.9	30.3	474.3	30.0	80.2	3.2	0.4	0.4	2.8
**Overall**	1410.5	2660.6	535	8303	531	1329	-	-	-	-

Table S4. Different genes present in the interval of AcB1-A6.1 locus in *B. juncea* Tumida and Varuna and syntenic regions in *A. thaliana*, *B. rapa* with the predicted gene functions in *A. thaliana*

Block	*A. thaliana*	*B. rapa*	Tumida	Varuna	Molecular/Biological function in *Arabidopsis*
L	-	Bra025412	BjuA024124	A06_g5118.t1	-
L	AT3G30300	Bra025413	BjuA024125	A06_g5117.t1	O-fucosyltransferase family protein
L	AT3G30380	Bra025415	BjuA024126	A06_g5114.t1	alpha/beta-Hydrolases superfamily protein
L	AT3G30390	Bra025416	BjuA024127	A06_g5113.t1	Transmembrane amino acid transporter family protein
L	AT3G30460	Bra025417	BjuA024128	A06_g5111.t1	RING/U-box superfamily protein
L	AT3G30530	Bra025418	BjuA024129	A06_g5109.t1	basic leucine-zipper 42, DNA binding
L	AT3G30580	Bra025419	BjuA024130	A06_g5108.t1	hypothetical protein
L	AT3G30725	Bra025420	BjuA024131	A06_g5106.t1	glutamine dumper 6 which is involved in amino acid transport
L	AT3G30775	Bra025421	BjuA024132	A06_g5105.t1	Methylenetetrahydrofolate reductase family protein
L	AT3G30841	Bra025423	BjuA024133	A06_g5104.t1	Cofactor-independent phosphoglycerate mutase
	-	Bra037476	BjuA046223	A06_g5047.t1	-
V	At5g48910	Bra037454/Bra037452	BjuA046213/BjuA046214	A06_g5024.t1/A06_g5023.t1	Pentatricopeptide repeat containing protein
	-	Bra037451	BjuA046215	A06_g5020	-
V	At5g48940	Bra037450	-	-	Leucine-rich repeat transmembrane protein kinase family protein
	-	Bra037449	-	-	-
	-	Bra037448	BjuA046216	A06_g5018.t1	-
	-	Bra037447	-	A06_g5014.t1,A06_g5016.t1	-
V	At5g48950	Bra037446	BjuA046219	A06_g5013.t1	Thioesterase superfamily protein
V	At5g48953	-	-	-	LCR86 (Low-molecular-weight cysteine-rich 86)
V	At5g48960	Bra037445	BjuA046220	A06_g5012.t1	HAD-superfamily hydrolase
	-	Bra037444	BjuA046221	A06_g5011.t1	-
V	At5g48970	Bra037443	BjuA046222	A06_g5008.t1	Mitochondrial substrate carrier family protein
V	At5g49010	Bra037442	-	A06_g5007.t1	SLD5 (synthetic lethality with dpb11-1 5); gins complex subunit 4
V	At5g49015	Bra037441	-	-	Expressed protein
V	At5g49020	Bra037440	-	A06_g5005.t1	Protein arginine methyltransferase 4A
V	At5g49030	Bra037439	-	A06_g5004.t1	tRNA synthetase class
V	At5g49060	Bra037438	-	A06_g5003.t1	DnaJ heat shock amino-terminal domain protein
V	At5g49100	Bra037437	-	A06_g5002.t1	vitellogenin-like protein
V	At5g49120	Bra037436	BjuA023852	A06_g5001.t1	DUF581 family protein, putative
V	At5g49130	Bra037435	BjuA023851	A06_g5000.t1	MATE efflux family protein
V	At5g49160	Bra010026	-	-	MET1 (methyltransferase 1)
	-	Bra037434	BjuA023853/BjuA023850	A06_g4999.t1	
	At5g49170	Bra037433	BjuA023849	A06_g4998.t1	Hypothetical protein
	At5g49190	Bra037432	BjuA023848	A06_g4997.t1	Sucrose synthase activity
W	At5g49210	Bra037430	BjuA023846	A06_g4995.t1	stress response NST1-like protein
W	At5g49215	Bra037429	BjuA023845	A06_g4994.t1	Pectin lyase-like superfamily protein
W	At5g49220	Bra037428	BjuA023844	A06_g4993.t1	Hypothetical protein

W	At5g49240	Bra037427	-	A06_g4992.t1	APRR4 (PSEUDO-RESPONSE REGULATOR 4) transcription factor
W	At5g49260	Bra037426	BjuA023843	A06_g4991.t1	Hypothetical protein
W	At5g49270	Bra037425	BjuA023842	A06_g4989.t1	SHV2 (SHAVEN 2) protein
W	At5g49280	Bra037422	BjuA023841	A06_g4988.t1	Hydroxyproline-rich glycoprotein family protein
W	At5g49300	Bra037421	BjuA023840	A06_g4987.t1	Zinc finger (GATA type) family protein
	-	Bra037420	BjuA023839	A06_g4985.t1	-
W	At5g49330	Bra037419	BjuA023838	A06_g4984.t1	AtMYB111 (myb domain protein 111); DNA binding / transcription factor
	-	Bra025426	-	-	-
	-	Bra025425	-	-	-
W	At5g49350	Bra010039	-	-	Glycine-rich protein family
W	At5g49360	Bra010038	BjuA023837/BjuA023836	A06_g4983.t1	BXL1 (BETA-XYLOSIDASE 1) hydrolase
W	At5g49400	Bra010037	BjuA023834	A06_g4981.t1	zinc knuckle (CCHC-type) family protein
W	At5g49420	Bra010027	-	-	MADS-box transcription factor family protein
W	At5g49430	Bra010036	BjuA023833	A06_g4980.t1	WD40/YVTN repeat and Bromo-WDR9-I-like domain-containing protein
W	At5g49450	Bra010035	-	-	AtbZIP1 (Arabidopsis thaliana basic leucine-zipper 1)DNA binding transcription factor
	-	Bra010034	-	A06_g4979.t1	-
W	At5g49510	Bra010033	BjuA023832	A06_g4978.t1	prefoldin 3
W	At5g49520	Bra010032	BjuA023831	A06_g4976.t1	WRKY DNA-binding protein 48
W	At5g49530	Bra010031	BjuA023829	A06_g4974.t1	SIN-like family protein
W	At5g49540	Bra010030	-	-	Rab5-interacting family protein

Table S5. List of the primers used for checking gene expression and nucleotide duplication

Primer	Sequence (5’ to 3’)
Var_F1	ATGAGAACGAACTCCAGGAGAA
Var_2R	CGCTTCAACCGTATATTTCTTGTTAGC
Var_stopF	CACTTCAACTGTCAATGCTCATACT
Var_stopR	CTCCAGGTTTTTTAATTCACGGAGAG
Tum046215_F1	CTATTGCTGGGAGAACGTAGACATTTA
Tum046215_R2	AGTATGAGCATTGACAGTTGAAGTG

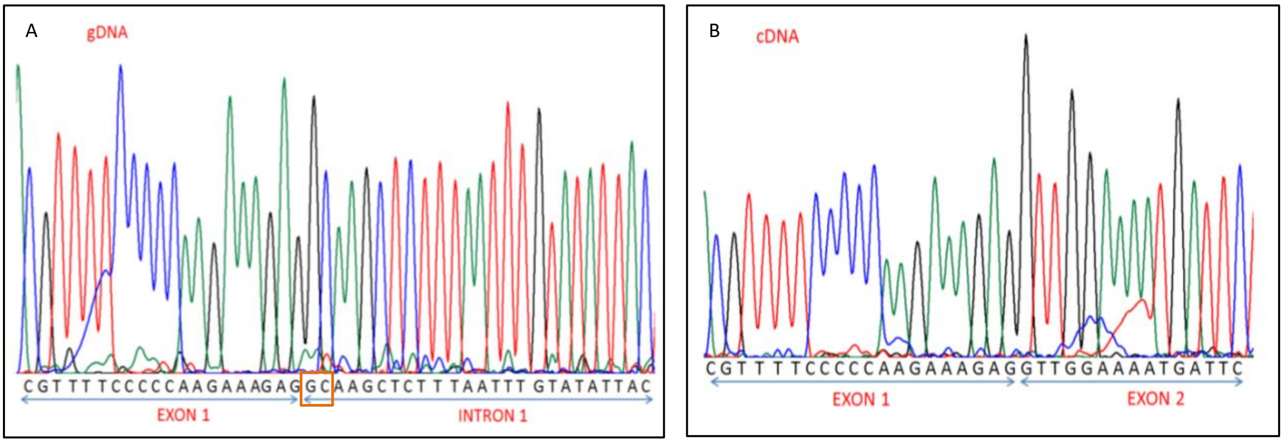

Fig. S1. Sequencing chromatogram of Tumida genomic DNA and cDNA. (A) Genomic DNA has a GC instead of GT at 5’end of the Intron-1 indicated by an orange box. (B) cDNA sequence in which the intron has been spliced out and only exons could be seen.

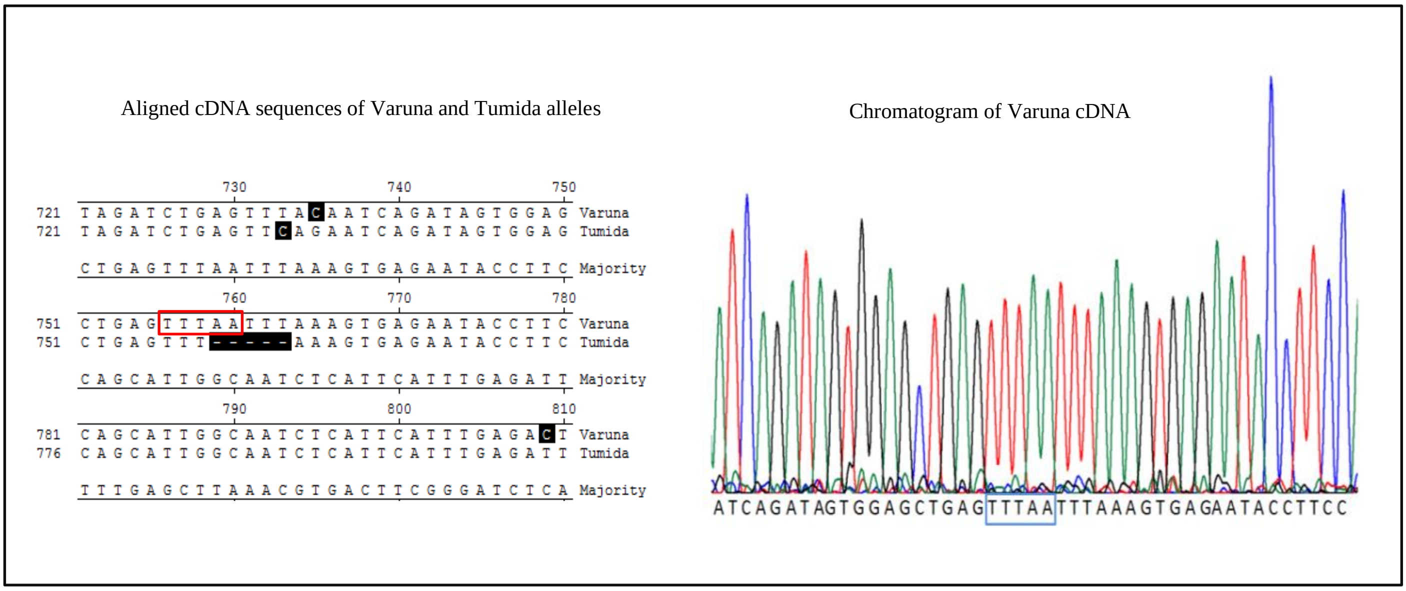

Fig. S2. Aligned cDNA sequences of susceptible and resistant alleles and chromatogram of Varuna cDNA. The aligned region and the Varuna cDNA showed the presence of a duplicated five bp sequence (TTTAA) in Exon 3 at the position 755-760 indicated with a red and the blue color box, respectively.

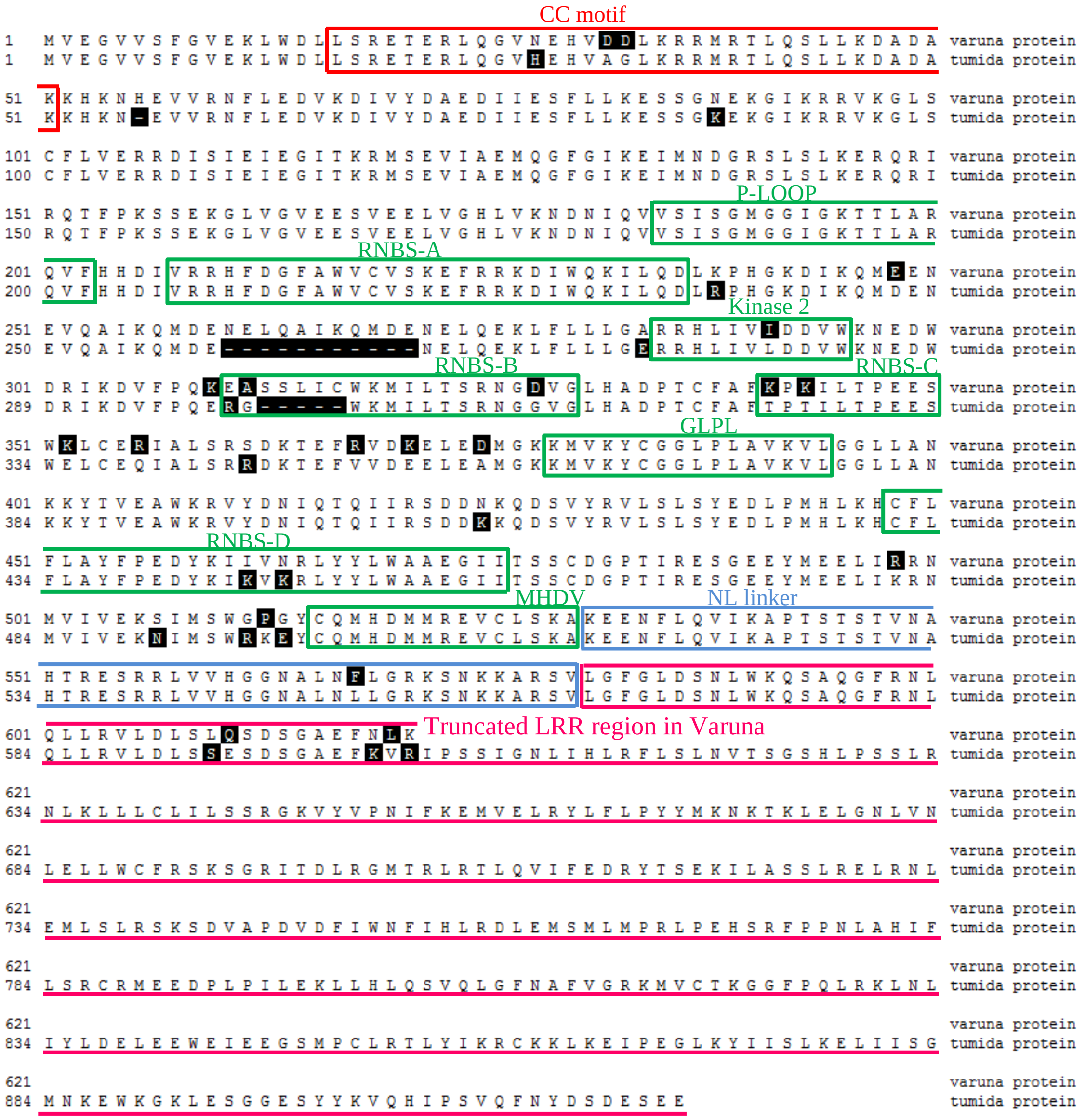

Fig. S3. Schematic representation of conserved motifs present in the protein sequences of susceptible (Varuna) and resistant (Tumida) allele of *BjuA046215* gene. The conserved motifs of coiled coil domain and NB domain including P-loop, RNBS-A, kinase 2, RNBS-B, RNBS-C, GLPL, RNBS-D, MHDV are highlighted with red and green colors respectively, whereas NL linker is represented with blue color. The premature truncation of polypeptide due to a frameshift in the ORF of the susceptible allele in Varuna has been highlighted in pink color.

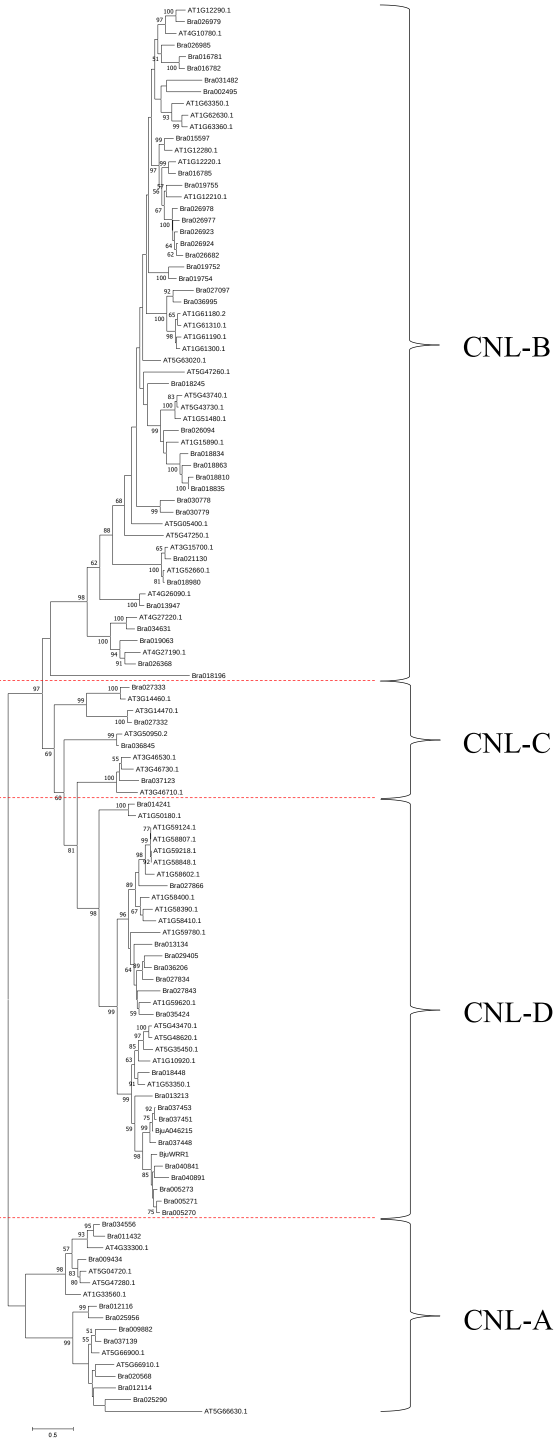

Fig. S4. Phylogenetic relationship of CNL type of NBS-encoding genes in *A. thaliana*, *B. rapa*, and the *B. juncea* genes – *BjuWRR1* and *BjuA046215* (*BjuWRR2*). The phylogenetic tree was constructed by MLM available in MEGA 7 software. Numbers on branches indicate the percentage of 1000 bootstrap replicates that support the adjacent node; bootstrap results were not reported if the support was <50%. Black braces at right indicate the subgroup names (CNL-A, CNL-B, CNL-C, and CNL-D); subgroups were defined as described by Meyers et al. (2003).
